# Dissecting and customising the Childhood Obesity Prevention Advisory Council (COPAC): the development and application of a community engagement framework to improve childhood obesity prevention among migrant populations

**DOI:** 10.1080/16549716.2017.1321822

**Published:** 2017-06-02

**Authors:** A. M. N. Renzaho

**Affiliations:** ^a^ Humanitarian and Development Studies, School of Social Sciences and Psychology, Western Sydney University, Penrith, NSW, Australia

**Keywords:** Community-based participatory research, community advisory council, obesity disparities, culturally diverse, obesity inequalities

## Abstract

**Background**: Migrant communities in Australia bear a disproportionate childhood obesity burden. They also show poor engagement in obesity prevention initiatives which may contribute to widening obesity disparities. Community engagement has been shown to be effective in reducing health disparities by improving migrant communities’ participation in prevention programmes.

**Objective**: This study aimed to develop a community engagement framework to improve childhood obesity prevention among migrants.

**Design**: Based on the African Review Panel model and the Community-Based Participatory Research conceptual logic model, the Childhood Obesity Prevention Advisory Council (COPAC) framework was developed and established in four disadvantaged areas in Victoria, Australia. The COPAC included service providers and migrant community members from the same project’s site.

**Results**: COPAC demonstrated several benefits including cross-organisational and multidisciplinary collaborations; understanding of the cultural barriers in childhood obesity prevention; enthusiasm from the COPAC members in addressing childhood obesity in their multicultural communities; equitable involvement, motivation, and empowerment of COPAC members in research development; and establishing organisational affiliations to foster long-term community involvement. This study also documented several challenges in community engagement including lack of prioritisation of migration-related childhood obesity disparities by the policymakers; staffing constraints among service providers leading to frequent disruptions in COPAC members’ contributions; and lack of adequate training and skill-building of bicultural workers.

**Conclusions**: The COPAC model adopted a flexible and dynamic community engagement process to suit the ongoing needs of the migrant community which incorporated the existing talents and resources within the community. For effective community engagement of migrant communities, it is important for policymakers to develop the knowledge, capacity and skills of the bicultural migrant workforce. Integrating both service providers and migrant community members in the COPAC has demonstrated that a multifaceted community-led approach has the potential to reduce childhood obesity-related disparities in Australia.

## Background

Data from the Australian Institute of Health and Welfare [1] indicate that obesity is a growing public health issue in Australia and globally, as well as the second-highest risk factor contributing to the burden of disease in Australia. In 2008, obesity was recognised as one of the nine national health priority areas [1]. A number of family and community-based obesity prevention programmes have been piloted and implemented over the last two decades [2,3]. The effectiveness of these programmes has been evidenced by the fact that childhood obesity prevalence is no longer increasing, with previous increases plateauing or even declining [4,5].

**Table 1.** Development and application of the COPAC.

Goals	Activities
**The development process of the COPAC model and membership**	
Recruitment of COPAC members	Identification of suitable professional stakeholders and ethnic community members
Establishment of the COPAC	Conducting community forums to inform potential COPAC members about childhood obesity prevention among migrant communities
Formalising the COPAC membership	Evidence of membership – memorandum of understanding
**Operationalisation of the COPAC: community readiness assessment**	
Participant recruitment	Community mobilisation of ethnic communities
Development of data collection tools	Ensuring the cultural appropriateness and understandability of the interview schedule
Interpretation of readiness assessment results	Clarification of unclear locally relevant community issues and providing explanations of any cultural meanings in ethnic respondent interviews
Dissemination of findings to the community	COPAC members functioning as channels of information dissemination in their respective services
**Application and policy implication: generation of recommendations**	
Environmental influences on healthy eating	Systems mapping of food environments
Community structure analysis	Document analysis and discussions to identify community structures for childhood obesity prevention initiatives
Barriers and facilitators to participation	COPAC group discussions on findings from readiness analysis
Improvements in service delivery	COPAC members feed back on readiness assessment

However, while effective in Anglo and non-disadvantaged communities, community-based interventions (CBIs) in multicultural and disadvantaged communities have not been effective to date (having no effect on body mass index) [6,7], and have relied on programme delivery through schoolchildren as agents of change [6,7]. It is possible the differential effects of these interventions in Anglo and non-disadvantaged communities reflect more agency by Caucasian children for personal and family action than by children from collective, hierarchical and authoritarian cultures [8,9]. It is likely that obesity prevention in non-Caucasian cultures requires theoretical frameworks that reflect the influences and complexity of children’s socio-economic, cultural and environmental contexts [8,9]. Even though they bear the greatest burden of obesity, many of these CBIs did not target individuals from migrant and low socio-economic backgrounds [3,6,7]. Migrant and disadvantaged communities remain under-represented in obesity research [10,11]; they are less likely to be invited to participate in research studies, notwithstanding a landmark systematic review demonstrating that in developed countries they are more likely to participate in research than are mainstream communities [12]. Moreover, health interventionists adopt community engagement approaches that work for mainstream populations but do not cater to the linguistic and cultural needs of migrant groups, resulting in failure to achieve programme outcomes [13,14]. For example, most randomised controlled trials under-recruit or even exclude migrants from non-English-speaking backgrounds in part because one of the inclusion criteria is the ability to speak English. This under-representation of migrant populations threatens the generalizability and the external validity of these interventions, because participants with any obesity risk factors who might otherwise benefit most from such interventions get excluded from the interventions’ evaluation [15,16].

The increased obesity risk, disproportionate obesity burden and poor engagement of migrant communities in obesity prevention services contribute to the widening of migration-related obesity inequalities in Australia [17–20]. Over the past few decades, community engagement has been increasingly recognised as a powerful tool for tackling health inequalities and harnessing community potential in the health improvement of disadvantaged populations [21,22]. A recent systematic review found that community engagement enhances migrant communities’ participation in health programmes [23].

Community-based Participatory Research (CBPR) is one community engagement approach which bridges the gap between research and practice by addressing power imbalances and enabling knowledge exchange, and has been extensively used among cross-cultural and disadvantaged settings [24,25]. CBPR has been found effective in eliminating health disparities through the equitable engagement of the community [25]. The existing literature shows that community engagement is a key component of effective and culturally competent childhood obesity prevention programmes [26,27]. However, community and family-based obesity interventions have often relied on community steering committees as the main method of community engagement. Community steering committees have involved community members, organizational representatives and researchers in all aspects of the research process. Nonetheless, community members sitting on these steering committees have predominantly been educated community gatekeepers, keen to protect their own personal interests and push personal agendas, which may be incompatible with the needs of their respective communities. Relying on gatekeepers as part of a community engagement model means that this approach fails to recognise the different overlapping identities and needs within the concerned communities and also fails to build on the strengths and resources within the community [28].

In Australia, a number of steering committees have been used as models of community engagement in childhood obesity prevention. These include the Obesity Policy Coalition, the Parent’s Jury as a platform to advocate for healthy food and environments for children, and the Food Alliance which addresses issues pertaining to food sustainability, security and equity [29]. As with similar previous initiatives, these community engagement strategies have focused on the most articulate members of the community, mainly the educated English-speaking professionals, but have excluded those who are most affected by the obesity epidemic. The exclusion of disadvantaged communities from these initiatives has been due to many factors including service providers and researchers not knowing how to engage the affected communities, poorly documented governance processes of community engagement, and the community engagement being process-oriented rather than outcome-oriented which often leads to consultation fatigue and community mistrust of the process [28,30,31]. Therefore, the aim of this study was twofold: (1) to trial an outcome-oriented and community-driven engagement framework to reduce migration-related obesity inequalities; and (2) to document and describe the dimensions involved in the development and application of the framework in childhood obesity prevention among migrant communities living in Australia.

## Methods

### Theoretical framework

The community-engagement framework was based on the CBPR conceptual logic model comprising four dimensions: context, group dynamics (including structural, relational and individual sub-dynamics), research design, and outcomes [25,32]. The model emphasises the equitable involvement of the community members in programme development processes to ensure the long-term sustainability of the advisory councils beyond the research-funding period. Since this model has the potential to reduce power imbalances and incorporate the cultural norms and beliefs of the community, it is appropriate for research and practice involving culturally and linguistically diverse (CALD) migrant communities.

### Setting

The Childhood Obesity Prevention Advisory Council (COPAC) community engagement framework was established in four purposively selected areas, each of which has an Index of Relative Socioeconomic Disadvantage (IRSD) score of < 1000, a cut-off score used to define socio-economic disadvantage [33]. The study’s areas were Hume (IRSD = 952), Greater Dandenong (IRSD = 895), Brimbank (IRSD = 926) and Maribyrnong (IRSD = 974). More than 50% of the population in these four areas originate from migrant or refugee backgrounds [34], hence providing the best environment to evaluate migrant communities’ readiness to engage with obesity prevention initiatives in Victoria, Australia. The study was approved by the Monash University Human Research Ethics Committee, approval no. CF 14/1443–2014000678.

### Data source and procedures

The COPAC community engagement framework was implemented in three complementary phases. Phase 1 focused on trialling the African Review Panel (ARP) as a community engagement model. In 2002, as part of the African Migrant Capacity Building and Performance Appraisal framework, the ARP was developed as a culturally competent tool for community engagement to guide programmes aimed at reducing childhood obesity among African migrants in Australia. African migrants were chosen as a case study for four reasons: (1) they have been a fast-growing cluster of the Australian population since the 1990s [35]; (2) they experience rapid weight gain following migration to Australia [36,37]; (3) they define obesity in positive terms, hence acting as a disincentive to participate in obesity prevention programmes and requiring a cultural approach as a key to tackling childhood obesity in these communities [36]; and (4) they predominantly come from a non-English-speaking background and have low health and obesity literacy [38–41].

**Figure 1.**
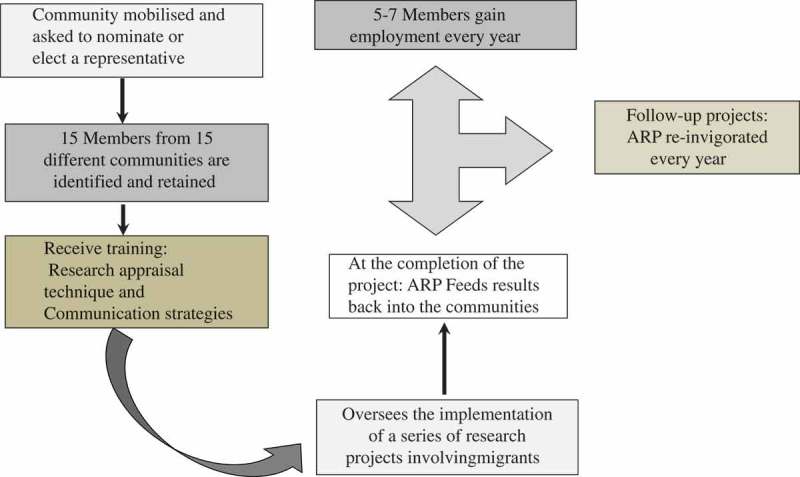
The process of engagement for the African Review Panel.

In establishing the ARP, it was recognised that ‘the opportunity arises for communities and science to work in tandem to ensure a more balanced set of political, social, economic, and cultural priorities, which satisfy the demands of both scientific research and communities at higher risk’ [42,p.31]. ARP members were either nominated by their respective communities or recruited for their knowledge and expertise through community health workers and African community networks. They were trained in the ethical conduct of research, approaches to research implementation (including the importance of ethics approvals, data collection techniques and the mapping of data analyses) and data dissemination strategies using bilingual workers. The panel comprised 15 members, predominantly from non-English-speaking backgrounds. Research team members became mentors of the panel members and provided them with job references to complement the training they received, hence providing employment opportunities. The process of engagement is summarised in Figure 1. From 2002 until 2013, the ARP oversaw the implementation of seven major projects, with a total budget of $1.8 million [38,40,43].

Phase 2 involved a systematic review exploring the role of community engagement in improving the health of disadvantaged populations [23]. The findings from the systematic review suggested that community engagement models can lead to improved health and health behaviours among disadvantaged populations so long as they are designed and implemented through effective community consultations and participation. The main characteristic of an effective and culturally competent community engagement model applicable among disadvantaged communities was the community readiness to engage. That is, such a community engagement model had the following components: engagement of community partners in all stages of research development, facilitating knowledge exchange between community and academic partners, and achieving balance between research and action [23].

Phase 3 examined the readiness of disadvantaged communities to engage with childhood obesity prevention initiatives [44] and barriers and facilitators to the engagement of migrant communities in obesity prevention initiatives [43]. Using the community readiness model, the community readiness study [44] found that disadvantaged communities had low levels of readiness to engage with the existing childhood obesity prevention initiatives, lacked knowledge of childhood obesity and its prevention, and reported facing challenges in initiating and sustaining participation in obesity prevention initiatives. Identified priorities to improve community readiness and reduce obesity-related disparities included addressing low obesity-related literacy levels, facilitating the capacity-building of bicultural workers to deliver obesity prevention messages, and integrating strategies addressing low obesity literacy and the capacity building of bilingual workers into existing Australian health policy and practice. In addition, the study barriers and facilitators to the engagement of migrant communities in obesity prevention initiatives [43] identified key barriers as being: competing priorities in the post-migration settlement phase; language, cultural and programme accessibility barriers; low levels of food and health literacy; excessive junk food advertisement targeting children; and lack of mandatory weight checks for schoolchildren. Key facilitators were having bicultural playgroup leaders; ethnic community groups; and school-based healthy lunchbox initiatives.

The implementation process of the aforementioned studies and emerging findings were used to operationalise the COPAC. That is, in order to operationalise the COPAC, members were recruited to oversee the implementation of the pilot studies and its effect on the community readiness to engage and the barriers and facilitators. The COPAC ensured that the research protocol was appropriately adapted to suit the culture of the local community and thus incorporated the voice and agency of the migrant community in its agenda. COPAC members provided input into the recruitment of study participants, the development of data collection tools, and the interpretation of results. Migrant COPAC members provided clarifications on culturally sensitive issues in the readiness assessment findings to enable a better understanding of the ethnic respondent interview data. They also facilitated the dissemination of findings to their respective ethnic communities through culturally appropriate channels such as ethnic committee meetings and ethnic festivals to raise awareness of childhood obesity among migrant communities. COPAC members who were service providers facilitated the dissemination of the readiness findings in their respective services including city councils, early years’ services, schools, community health centres and maternal and child health (MCH) services.

### Data analysis

A directed content analysis was used in this study. Directed content analysis starts with existing research findings, which are in turn then subjected to thematic coding to validate and extend a conceptual framework [45]. The first stage involved identifying key concepts and predetermined codes from the three phases that informed the COPAC as initial categories [45]. The next stage involved determining the operational definition for each category using individual themes as the unit for analysis. Since the analysis was based on the preliminary ARP, an initial list of coding categories from the model was generated, and these categories were modified within the course of the analysis as new concepts emerged inductively from the systematic review and pilot studies [43,46]. The last stage of analysis involved making sense of the identified themes and their properties and finalising the COPAC model.

## Results

### Integrating lessons learnt from ARP into the COPAC

**Figure 2.**
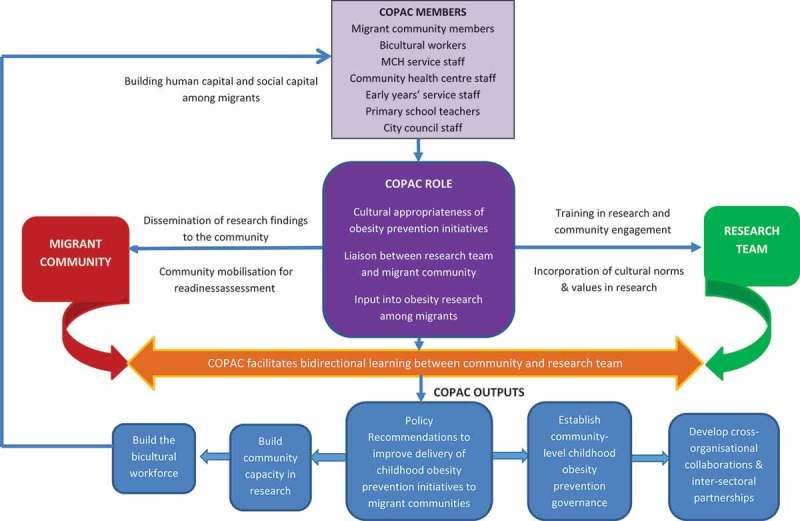
COPAC community engagement framework.

Funded by the Australian Research Council, the COPAC was modelled on the ARP which was expanded to include non-African migrants from various ethnicities to engage them in childhood obesity prevention research. At its inception, it was envisaged that incorporating the ‘voice’ of the community in designing engagement strategies would potentially address the barriers to engagement in obesity prevention initiatives. Therefore, the COPAC was structured as a community advisory council comprising diverse community members who were committed to effecting change including community champions [38,40]. Further, because COPAC members were involved in the co-development of key recommendations to improve the participation of migrant communities in existing obesity prevention initiatives, the study included service providers and policymakers in the COPAC model to ensure its sustainability and integration into policy and practice (Figure 2).

### The development process of the COPAC model

The nine CBPR principles proposed by Israel et al. [28] were used as operational guidelines for the implementation of the COPAC. These principles included:

1. *CBPR involves working with the community as a unit of identity*. In this study, the community was defined as a unit of identity based on their geographical location, since community health programmes are usually governed by the local city councils in each local government area (LGA). The COPAC aimed to work with existing communities in the LGA to strengthen the sense of community through communal engagement in order to improve the communities’ participation in the existing childhood obesity prevention initiatives.
2. *CBPR builds on strengths and resources within the community*. The COPAC aimed to identify and utilise the existing resources and strengths within the community including the talents and skills of ethnic community members; social networks based on trust-building; and structural assets including places of worship, ethnic community gatherings and schools.
3. *CBPR involves the collaborative and equal involvement of all partners in all phases of the research process*. The COPAC aimed to ensure the equal distribution of power among all its members, both service providers and ethnic community members, with regards to decision-making on the various aspects of the research process development of data collection tools, participant recruitment and interpretation of study findings.
4. *CBPR addresses locally relevant problems and considers multiple determinants of the problem*. According to this principle, the COPAC aimed to understand the immediate concerns of the migrant community regarding childhood obesity, its consequences and its prevention. COPAC also facilitated the analysis of various environmental influences impeding the adoption of healthy lifestyle behaviours among migrant communities.
5. *CBPR involves a co-learning and empowering process*. Being developed to improve the health inequalities among migrant communities, the COPAC aimed to facilitate the bidirectional transfer of knowledge between ethnic community members and service providers. Ethnic community members were equipped with knowledge of childhood obesity prevention and skills in healthy lifestyle behaviours while service providers gained knowledge of the cultural influences on child obesity from community members. This bidirectional knowledge transfer helped expose inequalities in the knowledge of programme delivery and programme perception among both groups, which highlighted the reasons for low or non-participation of migrant communities in the existing obesity prevention services.
6. *CBPR involves a cyclical and iterative process*. By involving stakeholders from existing services and ethnic community groups, the COPAC ensured the identification of childhood obesity as a priority for the community. The feedback from the community on research findings improved the adaptability of current childhood obesity prevention services in the target areas.
7. *CBPR achieves a balance between research and action*. COPAC aimed to contribute to the evidence base on engagement of migrant communities in childhood obesity prevention initiatives by exploring the cultural barriers affecting programme participation among these communities. This knowledge was incorporated into the development of tailored strategies. By having service providers from a range of services in its team, the COPAC showed the potential to facilitate the translation of research into policy especially at the local government level.
8. *CBPR disseminates findings and knowledge gained to all partners*. The presence of bicultural workers in the COPAC facilitated the dissemination of findings appropriate to the literacy levels of the community. This process also enabled migrant community ownership towards addressing the problem of childhood obesity.
9. *CBPR involves a long-term commitment by all partners*. By involving service providers who were already working within the current system as COPAC members, there was scope for sustainability in the obesity prevention efforts of the COPAC. Further, by lobbying for the training of bicultural playgroup facilitators and community leaders in healthy lifestyle behaviours, the COPAC showed the potential to create a long-term commitment towards childhood obesity prevention among migrant communities.

### The implementation process of the COPAC model and membership

#### COPAC membership and structure

COPAC members were purposively sampled covering professional stakeholders involved in the health and wellbeing of children, as well as ethnic migrant community members (Table 1). Through local city councils in the project areas, a list of health services for young children was established. These services included MCH services, early years’ services which included kindergartens, playgroups, family day-care and childcare facilities, community health centres, city councils, primary schools and refugee health services. The research team contacted professional stakeholders from these services initially through phone and email and followed up with face-to-face meetings to inform them about the study as well as to invite them to join the COPAC. Purposeful sampling allowed the research team to ensure COPAC members represented stakeholders at various levels of service delivery.

Four COPAC groups were established in total with one group per study area, in order for the engagement to be relevant to the local community. Ethnic COPAC members included those belonging to migrant communities, who were active in their community, with strong community networks and who possessed a reasonable knowledge of the various community activities organised at the local council. They were recruited through bilingual workers, the local city councils, as well as through bilingual playgroup facilitators in the community. Interested ethnic community members provided their contact details to the bilingual facilitators who in turn informed the research team. All interested stakeholders (professional and ethnic) were invited to attend a community forum organised by the research team. The forum involved presentations on the current situation of childhood obesity among migrant communities and the challenges associated with engaging migrant communities in childhood obesity prevention initiatives. Following attendance at the community forum, interested COPAC members were given a memorandum of understanding form to sign and membership that was renewable after every 12 months.

However, membership was standardised across the study sites such that each COPAC was made up of 10 people, with equal numbers of professional stakeholders and community representatives. Professional members included MCH nurse managers, population health managers, school wellbeing officers, school nurses, playgroup leaders, childcare managers, day-care staff, refugee health nurses, dieticians, health promotion practitioners and kindergarten and primary school teachers. Ethnic COPAC members included bicultural playgroup facilitators, ethnic community group leaders, bicultural workers from city councils, interpreters from MCH services, and migrant parents who are members of community groups. Ethnic COPAC members were from African, Indian, Vietnamese, Burmese, Middle-Eastern and Chinese backgrounds. These ethnicities are representative of the ethnic proportions of migrants and refugees living in the study areas.

#### COPAC meetings

The inaugural meeting involved a briefing session by the research team, where each COPAC member received an information pack containing a brief outline of the objectives and outcomes of the research study, the organisational structure and governance of the COPAC, a schedule of meetings, the list of COPAC members in each area and a document outlining the roles and responsibilities of COPAC members. COPAC members received training on CBPR principles, ethical aspects of the research project including informed consent, privacy and confidentiality of research data, and familiarisation with data management techniques. COPAC members were also briefed on community engagement approaches including their equitable involvement in all phases of the research project, sharing of responsibilities and ensuring maximum community participation.

Bimonthly COPAC meetings were organised in each of the 4 study areas in the local city council offices for 12 months. In alignment with CBPR principles, there was no formal leadership structure within COPAC groups and all members were given an equal position within the group. All COPAC meetings were facilitated by the research staff. The meeting agendas included the inventory of and discussions on current obesity prevention initiatives in the community, the participation of migrant communities in these initiatives and the various factors influencing their participation. The agendas were developed by the research team in consultation with the project steering committee. Bicultural workers served as interpreters for ethnic community members and migrant parents during the COPAC meetings.

#### Governance of the COPAC and outputs

The COPAC was overseen by a steering committee. The steering committee was comprised of university academics (members of the research team), policymakers, city council health and wellbeing staff, and service providers. The research steering committee was responsible for solving any problems arising from COPAC membership, meeting attendance and discrepancies between COPAC members and the research team such as any disagreement on the direction of the research project. At the first meeting of the steering committee, the role and membership of the COPAC were established. After deliberation, the steering committee established that the role of COPAC was to primarily serve as a liaison between the research team and the migrant community, ensure migrant communities’ involvement in research projects within their LGAs, and enable the cultural appropriateness of the research agenda in alignment with the cultural norms of the beneficiary communities. The expected outputs of the COPAC included (1) development of policy recommendations to improve childhood obesity prevention among migrant communities; (2) build migrant community capacity in research; (3) establish a community-level childhood obesity prevention governance body; (4) develop cross-organisational collaborations and inter-sectoral partnerships; and (5) build the bicultural workforce which in turn would enhance the human and social capital of migrant communities to ensure the sustainability of the COPAC.

## Discussion

The study trialled a community-driven community engagement framework to reduce migration-related obesity inequalities among migrants in Melbourne, Australia. Consistent with best practice [28,47], the development and implementation of the COPAC were guided by key principles in effective community engagement. It was important to recognise the community as a unit of individual and collective identities among ethnically diverse migrant communities across four geographical areas in order to maximise participatory decision-making by all COPAC partners. The COPAC emphasised the strengths, relationships and resources within the community. In doing so it supported and expanded existing social structures and social processes and facilitated collaborative partnerships that allowed the community to work harmoniously together to identify obesity-related health issues and devise strategies to improve the community’s wellbeing. Collaborative partnerships were achieved by including ethnic COPAC members who were active in their community, with strong community networks and a reasonable knowledge of the various community activities organised at the local council level. In addition, the COPAC was responsible for disseminating findings and knowledge gained by all partners in their community. Such an approach not only allowed the institutionalisation and integration of the knowledge and action for the mutual benefit of all stakeholders, it also promoted co-learning, coproduction relationships and an iterative process [28,48]. This level of knowledge exchange and coproduction relationships are key factors for facilitating the systems mapping of the food environment, enabling the identification of key barriers and facilitators to participation of migrant communities in childhood obesity prevention initiatives, and identifying priorities for improvement in the current delivery of childhood obesity prevention services within the target communities.

### Some benefits related to the implementation of the COPAC

The development and operationalisation of the COPAC resulted in several benefits. The research team identified that COPAC members were interested in community engagement due to three reasons: (1) they were enthusiastic about childhood obesity being addressed in their community since they realised that it was a growing problem which was not readily understood by migrant communities; (2) they perceived COPAC to be a viable and sustainable process capable of bringing funds into the community to address childhood obesity; and (3) they found the process empowering as they felt valued because their input and involvement were being sought at all stages of the research development. This approach of promoting the user and community coproduction of obesity-related community services beyond community engagement is consistent with best practice [48]. It promotes the sustainability of community engagement, facilitates cross-organisational and multidisciplinary collaborations and ensures equitable community involvement in the partnership.

The user and community coproduction of obesity-related knowledge was multidimensional. For example, during the COPAC meetings, it was identified that MCH services were not equipped with adequate pictorial healthy eating resources and updated diet charts. Consequently, COPAC members who were health promotion practitioners from community health centres organised the delivery of more appropriate resources to the MCH services. This sharing of information and resources facilitated synergy and integration of efforts to address obesity across the study’s sites. Similarly, school wellbeing officers highlighted the lack of nutrition information sessions in primary schools. This problem was addressed by the COPAC members who were dieticians. They took steps to organise annual healthy eating seminars in their local primary schools. Evidence shows that developing collaborations between organisations is one of the key benefits of using community engagement in achieving positive study outcomes [49,50].

The promotion of the user and community coproduction of obesity-related knowledge and community services enhanced the applicability of the COPAC to real-world multicultural settings. Ethnic COPAC members enabled the identification of cultural beliefs of migrant communities towards childhood obesity prevention. For instance, MCH nurses perceived that low attendance at MCH services by migrant mothers was due to language barriers and could be addressed by the provision of interpreter services. However, ethnic COPAC members identified that migrant communities often followed dietary advice from family elders on food choices and could not understand the importance of MCH nurses providing dietary advice for children. The role and importance of elders in influencing food choices and preferences as well as health behaviours among migrant families at the expense of the advice provided by health professionals are well documented [51]. The COPAC framework helped bridge these cultural barriers between service providers and migrant communities.

Across all four study areas, the COPAC identified that childhood obesity prevention was not a priority for health services catering for the needs of migrant communities, with issues such as immunisation, domestic violence and infectious disease surveillance taking precedence over healthy lifestyle initiatives. The existing literature shows that lack of goal sharing and consensus on priorities for funding among policymakers is a key reason for non-effective community engagement [52]. Policymakers and service providers’ lack of interest in lifestyle-related diseases among migrants can often be exacerbated by migrants’ numeric disadvantage (value for money) and their low health literacy. For example, notes from COPAC briefing sessions and evidence from previous research suggest underfunding of migration-related research and health services [52]. In addition, the research shows that many migrants from non-English backgrounds do not consider childhood obesity to be a problem in their communities. Such a position is driven by the positive value migrants attach to obesity (e.g. large body equating to prosperity and post-migration success) and low obesity literacy [9,36,40].

The COPAC allowed the identification of the aforementioned cultural barriers and the best way to address them. For example, ethnic COPAC members stated that the existing cultural misconceptions on childhood obesity, its causes and consequences prevailing among migrant communities need to be addressed prior to expecting migrant communities’ involvement in the development of obesity prevention strategies and initiatives. Such a recommendation implied that improving obesity literacy among migrants should be a priority for service providers and policymakers. This observation, together with the known inverse relationship between health literacy and childhood obesity [53], suggests that systematic approaches to integrating health literacy and obesity prevention strategies may mitigate the growing burden of migration-related obesity inequalities.

### Some challenges related to implementation of the COPAC

The development and operationalisation of the COPAC among migrant communities involved certain challenges. Due to staffing constraints in childhood obesity prevention services in disadvantaged areas, service providers faced difficulties in maintaining regular attendance at COPAC meetings. Community health centres were short-staffed which led to poor attendance by dieticians and other allied health workers who were members of the COPAC, many of whom were part-time and on short-term contracts due to lack of adequate funding. It is well documented that empowering ethnic communities without corresponding improvements in the health system’s infrastructure can easily compromise the benefits of community engagement [30]. Therefore, the COPAC was flexible and implemented a fluid process that allowed movement between different types of engagement throughout the project cycle, with dieticians and health workers allowed to attend COPAC meetings on a rotational basis.

In order to address difficulties in updating new COPAC memberships due to staffing changes posed by short-term contracts, the COPAC developed clear handover guidelines to ensure continuity of engagement. Measures put in place to ensure the sustainability of the COPAC included individual orientation meetings and training sessions for new COPAC members on the community engagement process prior to COPAC meetings to ensure the seamless continuation of the engagement activities. According to Israel et al. [47], having the ‘right people around the table’ ensures the sustainability of the engagement process and achievement of the study’s goals. Therefore, involving organisations rather than individual members in our recruitment strategy proved useful in maximising the institutionalisation of the COPAC and having the right people around the table during COPAC meetings.

Another challenge was the low health literacy of bicultural playgroup facilitators. At the start of the project, no formal mechanism of training or skill-building of bicultural workers by the health services or community services existed in the four project’s sites. The COPAC mandated training active members of the migrant community to become community champions and volunteers in community health programmes. However, this recommendation was affected by the lack of a clearly defined structure and centralised governance in the current childhood obesity prevention services. The lack of coordination posed challenges for service providers in understanding their roles and avoiding duplication of services. The implementation of the COPAC demonstrated that, in order to address migration-related obesity inequality, policymakers need to consider a shift in the current obesity prevention governance from a ‘health system’ approach to a more ‘community-based’ one with equal input from all key stakeholders. This observation is consistent with key remarks from using a systems approach to tackle obesity in the United Kingdom. Some of the lessons from the United Kingdom suggest that there is a lack of a shared understanding of what systems approaches entail; systems approaches are hard to implement and require tools and guidance which are yet to be developed and tested; and their impact is difficult to establish due to the unpredictability of systems change [54].

## Conclusion

This study has found the COPAC to be a feasible community engagement approach to address childhood obesity prevention among migrant communities. Various lessons can be drawn. Firstly, by being both evidence-based and practice-based, the COPAC adhered to CBPR principles, hence demonstrating its wide applicability in real-world multicultural settings. Secondly, COPAC targeted available community-level channels for obesity prevention by involving multidisciplinary stakeholders capable of modifying the various multilevel determinants influencing childhood obesity. Thirdly, the operationalisation of the COPAC involved a balance between adhering to the research protocol and flexibility to accommodate the needs of the community. Especially with migrant communities being diverse in their culture and values, having a clear understanding of their receptivity prior to commencing community engagement was very important. Fourthly, the implementation of the COPAC involved a dynamic process which was adaptive to suit the ongoing demands of the migrant communities, prioritised the recognition of the knowledge and talents of ethnic community members, and fostered real power sharing and knowledge transfer. By utilising the skills of ethnic community members, not only was the COPAC able to understand challenges faced by migrant communities in accessing obesity prevention initiatives, it was also able to identify areas of improvement in the delivery of obesity prevention services to these communities. Finally, fostering organisational affiliations and commitment rather than individuals’ was a viable channel to developing sustainable community partnerships. With adequate funding and infrastructure support, such partnerships have the potential of developing culturally competent and community-owned obesity prevention initiatives.

## Paper context

Childhood obesity levels are rising among migrant groups due to their poor engagement in obesity prevention initiatives and cultural drivers. This paper describes the development of a community engagement model, the Childhood Obesity Prevention Advisory Council (COPAC), to improve childhood obesity prevention among migrant communities. Including both service providers and migrant community members as equal partners in community engagement, and improving the health literacy and skill-building of bicultural workers can ensure adequate migrant engagement in childhood obesity prevention initiatives.
